# Description of *Seba longimera* sp. nov. from hydrothermal vents in the Okinawa Trough, Northwest Pacific (Amphipoda, Amphilochoidea, Sebidae)

**DOI:** 10.3897/zookeys.899.39442

**Published:** 2019-12-12

**Authors:** Yanrong Wang, Chaodong Zhu, Zhongli Sha, Xianqiu Ren

**Affiliations:** 1 Key Laboratory of Zoological Systematics and Evolution, Institute of Zoology, Chinese Academy of Sciences, Beijing 100101, China Institute of Zoology, Chinese Academy of Sciences Beijing China; 2 Institute of Oceanology, Chinese Academy of Sciences, Qingdao 266071, China Institute of Oceanology, Chinese Academy of Sciences Qingdao China; 3 Laboratory for Marine Biology and Biotechnology, Qingdao National Laboratory for Marine Science and Technology, Qingdao, China University of Chinese Academy of Sciences Beijing China; 4 Center for Ocean Mega-Science, Chinese Academy of Sciences, Qingdao 266071, China Qingdao National Laboratory for Marine Science and Technology Qingdao China; 5 College of Biological Sciences, University of Chinese Academy of Sciences, Beijing 100049, China Center for Ocean Mega-Science, Chinese Academy of Sciences Qingdao China

**Keywords:** Taxonomy, Sebidae, new species, hydrothermal vents, Okinawa Trough

## Abstract

*Seba longimera***sp. nov.**, of the family Sebidae Walker, 1908, is described from hydrothermal vents in Okinawa Trough. Other two congenic species, *S. bathybia* Larsen, 2007 and *S. profundus* Shaw, 1989, are also reported from these hydrothermal vents, but the new species can be readily distinguished from them in having the merus of pereopods 5 and 6 extending beyond distal margin of carpus, coxa 4 smaller than coxae 2 and 3, and coxa 5 with the posterior lobe larger than the anterior one, rather than equilobate.

## Introduction

The genus *Seba* Spence Bate, 1862 currently contains 24 species (updated by Köppen and Coleman 2011), and occurs in the Mediterranean Sea, Eastern and Southern Atlantic Ocean, southern United States, Hawaiian Islands, Indian Ocean, Antarctica, Japan, Eastern Pacific, and Australia from shallow to deep waters (Larsen 2007; Ariyama 2009; Yerman and Coleman 2009). When the Chinese research vessel “KEXUE” surveyed the biodiversity of hydrothermal vents in Okinawa Trough in the western Pacific in 2014, some individuals referred to *Seba* were collected. After careful examination, those specimens exhibited some distinctive characters differentiating them from the other described *Seba* species. The new species is most similar to *S. bathybia* Larsen, 2007 and *S. profundus* Shaw, 1989, which are also reported from hydrothermal vents. However, *Seba longimera* sp. nov. differs from above two species in having pereopods 5 and 6 with the merus extending beyond the distal margin of carpus, coxa 4 smaller than coxae 2 and 3, and coxa 5 having the posterior lobe larger than the anterior one, rather than equilobate. The present work describes this new species and compares it with closely related species.

## Material and methods

The present material was collected by ROV “FAXIAN” during expeditions to the Okinawa Trough hydrothermal vents by the Institute of Oceanology, Chinese Academy of Sciences (**IOCAS**) in April 2014. All the specimens examined are deposited in the Marine Biological Museum, Chinese Academy of Sciences (**MBMCAS**), Qingdao, China. Specimens were examined and dissected under a dissecting microscope (ZEISS Discovery V20). Line drawings were prepared with a graphics tablet using Adobe Photoshop CS6 software. Length measurements are made along the outline of the animals, beginning from the anterior margin of head to the end of the telson.

**Figure 1.**
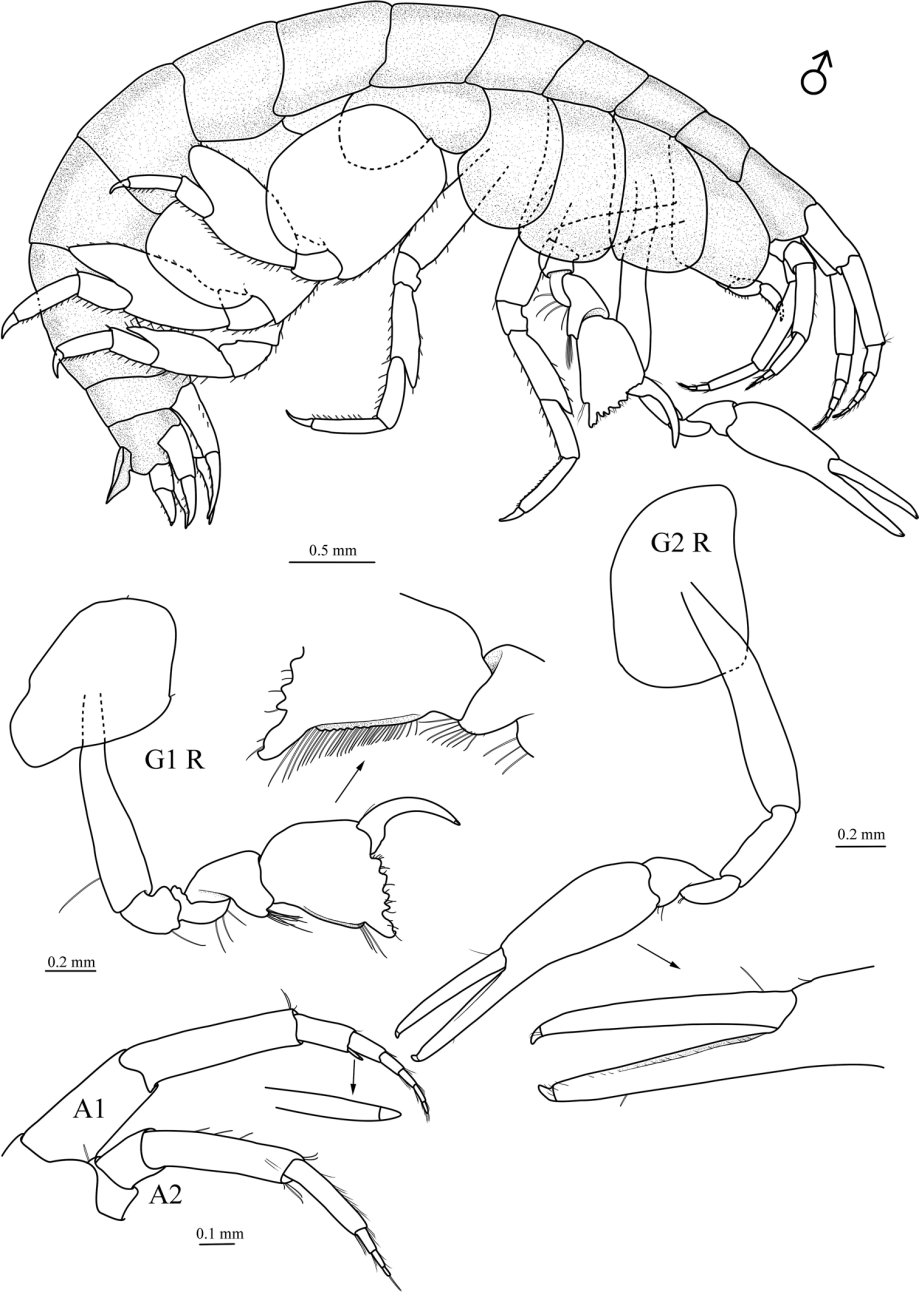
*Seba longimera* sp. nov., male holotype (6.1 mm) (MBM 286557), Okinawa Trough. G1 R, right gnathopod 1; G2 R, right gnathopod 2; A1, antenna 1 (with accessory flagellum enlarged); A2, antenna 2.

**Figure 2.**
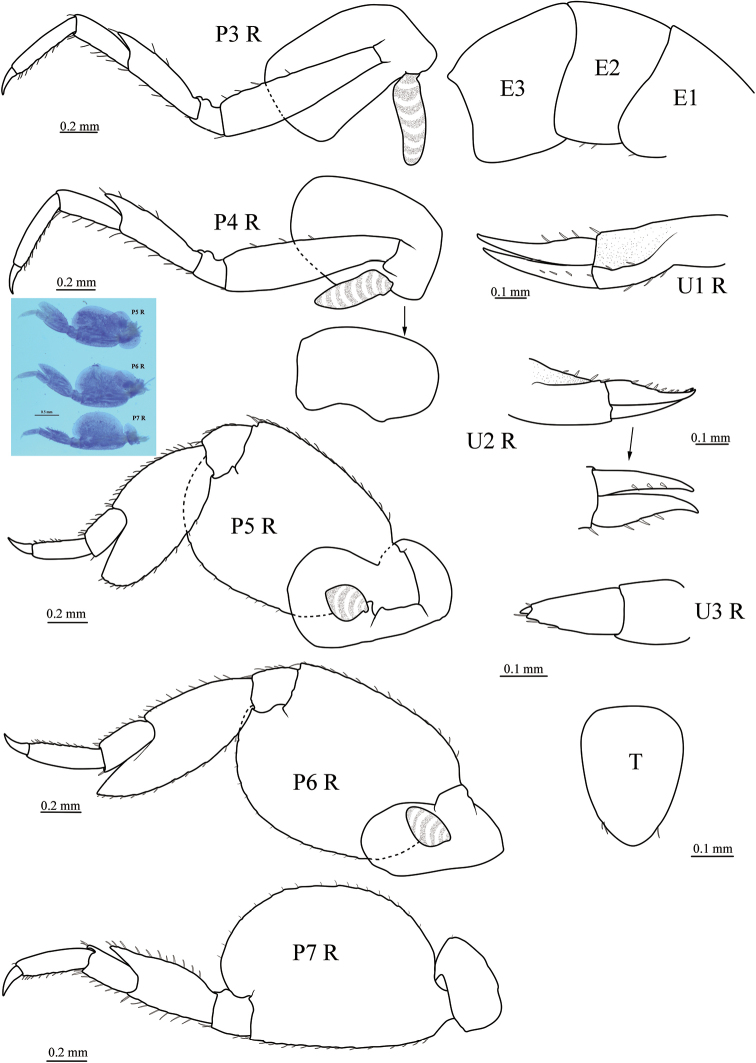
*Seba longimera* sp. nov., male holotype (6.1 mm) (MBM 286557), Okinawa Trough. P3 R, right pereopod 3; P4 R, right pereopod 4; P5 R, right pereopod 5; P6 R, right pereopod 6; P7 R, right pereopod 7; U1 R, right uropod 1; U2 R, right uropod 2; U3 R, right uropod 3; T, telson; E1–3, epimeron 1–3.

**Figure 3.**
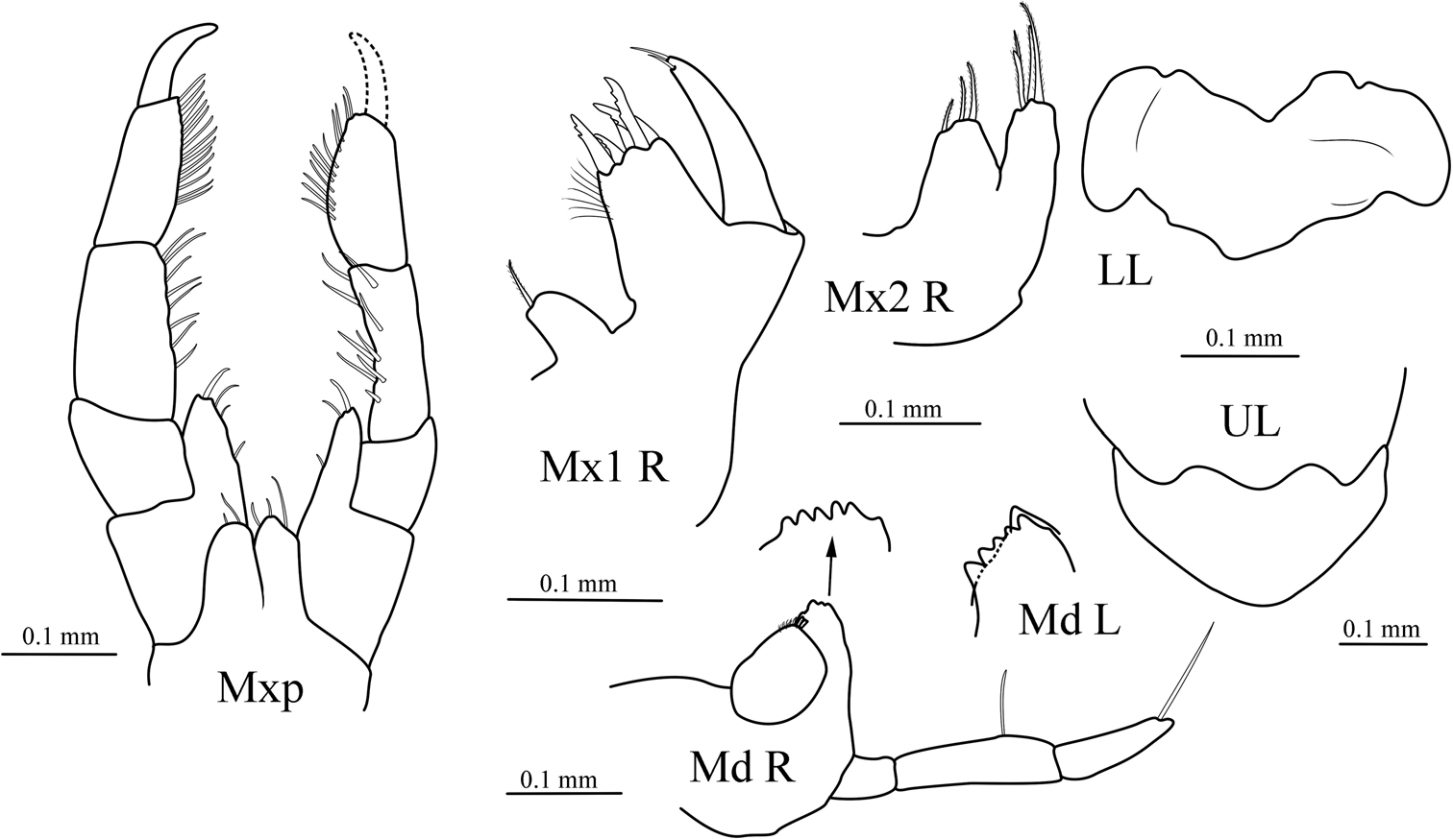
*Seba longimera* sp. nov., male holotype (6.1 mm) (MBM 286557), Okinawa Trough. UL, upper lip; LL, lower lip; Md R, right mandible (with incisor process enlarged); Md L, incisor process and lacinia mobilis of left mandible enlarged; Mx1 R, right maxilla 1; Mx2 R, right maxilla 2; Mxp, pair of maxillipeds.

**Figure 4.**
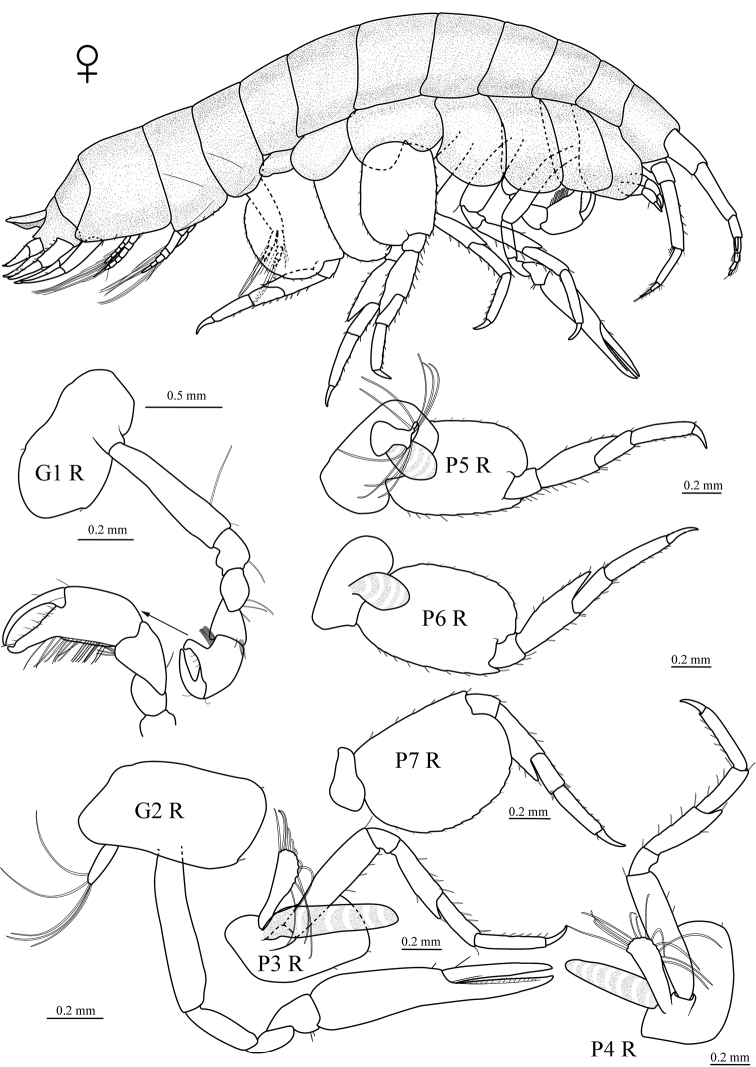
*Seba longimera* sp. nov., female paratype (4.5 mm) (MBM 286557), Okinawa Trough. G1 R, right gnathopod 1; G2 R, right gnathopod 2; P3 R, right pereopod 3; P4 R, right pereopod 4; P5 R, right pereopod 5; P6 R, right pereopod 6; P7 R, right pereopod 7.

The following abbreviations are used in Figures 1–4: A, antenna; E, epimeron; G, gnathopod; L, Left; LL, lower lip; Md, mandible; Mx1, maxilla 1; Mx2; maxilla 2; Mxp, maxilliped; P, pereopod; R, right; T, telson; U, uropod; UL, upper lip.

## Systematics

### Order Amphipoda Latreille, 1816

#### Suborder Amphilochidea Boeck, 1871

**Superfamily Amphilochoidea Boeck, 1871**

**Family Sebidae Walker, 1907**

**Subfamily Sebinae Holsinger & Longley, 1980**

##### Genus Seba

Taxon classificationAnimaliaAmphipodaSebidae

Spence Bate, 1862

1DA9D2E4-A3B8-5959-95E6-C707B3BF668B

###### Diagnosis.

See Ariyama (2009).

##### Seba longimera sp. nov.

Taxon classificationAnimaliaAmphipodaSebidae

14148D33-3870-568D-A978-20E48DE38F85

http://zoobank.org/BE6D0C6C-3BBE-46FB-9D21-21ECD4E39801

Figures 1 , 2 , 3 , 4

###### Material examined.

***Holotype***: male (6.1 mm) (MBM 286557), dissected, Okinawa Trough, 27°32'N, 126°58'E, RY0067, ROV-3, depth 1243 m, 16 Apr. 2014. ***Paratype***: female (4.4 mm), dissected, same data as holotype.

###### Additional materials.

1 female (4.5 mm), 3 males (4.2–5.5 mm) (MBM 286557), Okinawa Trough, 27°32'N, 126°58'E, RY0067, ROV-3, depth 1243 m, 16 Apr. 2014. 1 female (3.3 mm) 1 male (4.1 mm) (MBM 286560), Okinawa Trough, 27°33'N, 126°58'E, RY0051, ROV-3, depth 1243 m, 16 Apr. 2014. 15 females and males (<5.5 mm) (MBM 286565), Okinawa Trough, 27°33'N, 126°58'E, RY0069, ROV-3, depth 1243 m, 16 Apr. 2014.

###### Description of male holotype.

***Head*.** Eyes not visible in ethanol material. *Antenna 1* subequal in length to antenna 2; peduncular article 1 shorter than article 2 (0.8×); article 2 elongate, length 4.1× width; article 3 less than half the length of article 1; primary flagellum 5-articulate, not setose; accessory flagellum hardly reaching to end of 1^st^ flagellar article, 2-articulate, distal article tiny. *Antenna 2* with peduncular article 4 1.7× longer than article 5; peduncular article 5 much narrower than article 4; flagellum 3-articulate, not setose.

***Mouthparts*.** Epistome separate, upper lip rounded. *Right mandible* incisor well developed, with blunt denticles; palp 3-articulate, article 2 1.2× longer than article 3, bearing one long seta, article 3 bearing apical long seta; left mandible with dentate lacinia mobilis. *Lower lip* with inner lobes absent; mandibular lobes weak. *Maxilla 1* with inner plate rounded, bearing single robust apical seta; outer plate broadly truncate, bearing five robust apical setae; palp 1-articulate, with two apical setae. *Maxilla 2* with inner plate wider and shorter than outer plate, with 3 apical setae; outer plate with four apical setae. *Maxilliped* with very short inner plates, reduced to small lobes; outer plates short, slightly beyond distal margin of 1^st^ palp article, rounded, bearing few marginal and apical setae; palp 4-articulate, inner margins of article 2 and 3 bearing setae, dactylus elongate, slender.

***Pereon*.***Coxae 1–4* longer than broad, overlapping. *Gnathopod 1* parachelate, stouter than gnathopod 2; coxa with a small seta at posteroventral corner; basis linear, with 1 long seta on posterior margin; ischium subquadrate; merus subequal in length to ischium, posterior margin bearing three long setae; carpus shorter than palm, distally expanded, posterior margin lobate, bearing patch of long setae distally; palm slightly longer than deep, ventral margin fringed with long setae, with five bumps and few setae; dactylus curved, tapering. *Gnathopod 2* elongate, chelate; coxa oval; basis linear, naked; ischium longer than menus, naked; merus subequal in length to carpus; carpus distally expanded; propodus slender, narrowing distally, more than 4× longer than carpus. Lower finger of chela straight, bearing row of short setae on palmar edge; dactylus slender, straight, fitting palm, distally with few short setae. *Pereopod 3* with coxa slightly longer than coxa 2; basis not expanded posteriorly, anterior margin bearing four shorter setae in distal half length; ischium subrectangular, anterior margin notched; merus anterodistally drawn out, pointed, both anterior and posterior margins bearing few robust setae; carpus shorter than merus; propodus slightly longer than carpus; dactylus curved, tapering. *Pereopod 4* slightly larger than pereopod 3, but of similar appearance; coxa smaller than coxa 3, posterior margin excavated. *Pereopod 5* coxa bilobed, posterior lobe longer and larger than anterior lobe; basis evenly expanded, posteroproximal margin overlaps posterior coxal lobe; merus expanded, produced posteroventrally, well overreaching distal margin of carpus; carpus shorter than propodus; dactylus curved. *Pereopod 6* larger than pereopods 5 and 7, of similar appearance with pereopod 5, coxa unilobate. *Pereopod 7* smaller than pereiopod 5, coxa unilobate, much smaller than coxa 6; merus expanded, but smaller than that of pereopods 5 and 6, not extending past distal margin of carpus; carpus shorter than propodus.

***Gills*** present on coxae 3–6, small, not pleated.

***Pleon*.***Epimeron 1–3* smooth, posteroventral margin rounded. *Uropod 1* peduncle subequal in length to rami, with two distal, two lateral, and three medial robust setae; outer ramus slightly shorter than inner ramus, both outer and inner ramus with three robust setae. *Uropod 2* extending as far as uropod 1, peduncle slightly longer than rami, with one distal and five marginal setae; outer ramus slightly shorter than inner ramus; outer and inner ramus each with three marginal robust setae. *Uropod 3* uniramous, peduncle naked; ramus longer than peduncle, with two marginal setae and two setae at base of minute terminal article. *Telson* entire, laminar, tapering distally, smoothly rounded, distinctly overreaching end of uropod 3 peduncle, with one or two distolateral setae on each margin.

###### Sexually dimorphic characters.

Based on female paratype, 4.5 mm.

Gnathopod 1 parachelate, but tending to chelate; propodus much narrower than that of male; palm nearly straight, only bearing few setae. Pereopods 5 and 6 with basis not as expanded as in male, narrower than that of pereopod 7; merus not as expanded as in male, and not extending to distal margin of carpus.

###### Variation.

In one small male specimen (4.2 mm), the merus of pereopods 5 and 6 does not overreaching distal margin of carpus.

###### Etymology.

From the Latin *longus* (= long), referring to the merus of pereopods 5 and 6 overreaching the distal margin of carpus.

###### Distribution.

Northwest Pacific, Okinawa Trough, the hydrothermal vents at a depth of 1243 m.

###### Remarks.

The new species, reaching a length of 6 mm, is larger than all described *Seba* species that are usually less than 4 mm (Shaw 1989). *Seba longimera* sp. nov. is most similar to *S. bathybia* and *S. profundus*, which also are associated with hydrothermal vents, but it differs from these two species in the following characters. It differs from *S. bathybia* by having: the denticulate palm of gnathopod 1; coxa 2 lacking a notch, and coxa 4 smaller than coxae 2 and 3; the posterior lobe of the bilobed coxa 5 larger than the anterior lobe, and the expanded merus of pereopods 5 and 6 distinctly overreaching the distal margin of the carpus in male. Similarly, the new species differs from *S. profundus* by having coxa 4 smaller and the posterior lobe of coxa 5 larger than the anterior one; pereopod 6 larger than pereopods 5 and 7, rather than smaller than them as shown in Shaw (1989: fig. 3A); and the merus of pereopods 5 and 6 expanded beyond the distal margin of the carpus in male.

## Supplementary Material

XML Treatment for Seba

XML Treatment for Seba longimera
